# Subjective Cognition is Related to Patient-Reported Symptom Distress and Work Productivity Among Liver Transplant Recipients

**DOI:** 10.3389/ti.2023.10863

**Published:** 2023-01-17

**Authors:** Dami Ko, Sheila H. Ridner, Katherine A. Gifford

**Affiliations:** ^1^ School of Nursing, Bouvé College of Health Sciences, Northeastern University, Boston, MA, United States; ^2^ School of Nursing, Vanderbilt University, Nashville, TN, United States; ^3^ Vanderbilt Memory and Alzheimer’s Center, Department of Neurology, Vanderbilt University Medical Center, Nashville, TN, United States

**Keywords:** cognitive function, subjective cognition, patient-reported outcomes, symptom distress, work productivity, liver transplant recipients

## Abstract

Cognitive decline may prevent liver transplant (LT) recipients from staying healthy and independent. This study examined associations of objective and subjective, rated by LT recipients and caregivers, cognitive decline with patient-reported physical and psychological symptom distress, ability to perform household tasks, and workplace productivity among LT recipients. Sixty pairs of LT recipients and caregivers participated in this cross-sectional study. Subjective cognition was measured by the Everyday Cognition. Objective cognition was assessed with four cognitive tests, including the Repeatable Battery for the Assessment of Neuropsychological Status. Patient-reported outcomes were assessed with the Rotterdam Symptom Checklist-Modified, Profile of Mood States-Short Form, Creative Therapy Consultants Homemaking Assessment, and Work Limitations Questionnaire. Linear regression analyses related objective and subjective cognition to the patient-reported outcomes. While objective cognitive decline was not associated with any patient-reported outcomes, subjective cognitive decline was significantly associated with the outcomes. Higher LT recipient self-rated cognitive decline was associated with higher physical symptom distress ( β = 0.30, *p* = 0.006) and workplace productivity loss ( β = 14.85, *p* < 0.0001). Higher caregiver-rated cognitive decline was associated with lower household tasks performance ( β = −18.55, *p* = 0.015). Findings suggest to consider subjective cognition when developing an individualized post-transplant care plan.

## Graphical Abstract

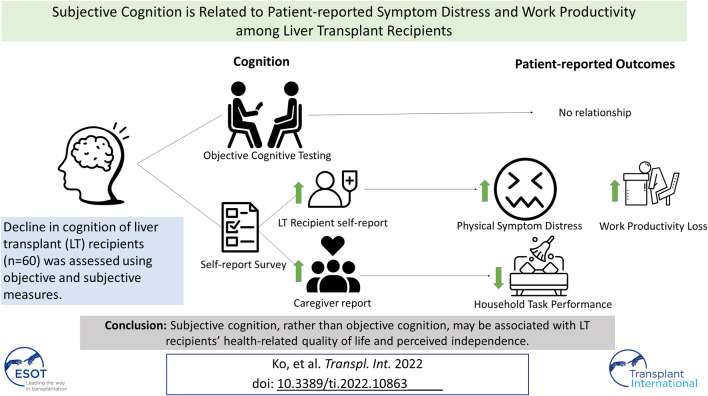

## Introduction

As more than 80% of liver transplant (LT) recipients survive beyond 5 years post-transplant (1), maintaining healthy and independent lives has become one of the top priorities for post-LT care (2). While some LT recipients tend to carry out activities to stay healthy and independent after LT, such as taking medication as prescribed or returning to employment, others do not. Up to 75% of LT recipients are non-adherent to their medication regimen (3–5), and less than 60% engage in either non-paid (e.g., homemakers or students) or paid work (6). Cognitive decline could be one of the potential factors that prevent successful performance of such activities. In fact, 9%–56% of LT recipients have objective impairment on formal cognitive testing (7–9).

Cognitive health is an important consideration for post-transplant recipients as cognitive decline after LT may affect recipients’ abilities to maintain healthy and independent lives. Intact cognition, particularly in memory, attention, and executive function, is required for successful performance of tasks necessary to maintaining overall health and independence (10). For example, LT recipients should perform health maintenance tasks, such as monitoring and managing symptoms and side effects of immunosuppressants. However, recipients with cognitive decline in the above areas may have reduced abilities to monitor and manage symptoms (11, 12) that potentially lead to physical and psychological symptom distress. Furthermore, decline in cognition, including the aforementioned domains, may reduce work capacity and productivity (13, 14). Recipients with cognitive decline may not be able to successfully perform household tasks, return to work, or stay employed after LT. While cognitive health appears to relate to physical and psychological symptom distress, ability to perform household tasks, and workplace productivity, there is a paucity of literature examining these associations. Understanding cognitive decline in relation to these patient-reported outcomes may expand our knowledge regarding overall wellbeing and disease burden associated with cognitive decline in this population. Further, such knowledge may also inform how clinicians can facilitate LT recipients in achieving healthy and independent lives.

This study investigated the associations between cognition and patient-reported outcomes of physical and psychological symptom distress, ability to perform household tasks, and workplace productivity among LT recipients. Objective and subjective measures of cognition (global and multiple specific domains) were assessed in relation to the patient-reported outcomes. We included subjective cognitive measures, reported by LT recipients and caregivers, to assess the feasibility of using these self-report cognitive measures given the ease of use in clinical practice (15). We included caregivers in this study since caregivers may estimate LT recipients’ cognition differently than recipients themselves (15). Subjective cognition reported by LT recipients and caregivers could be variously associated with patient-reported outcomes. In this study, caregivers were queried about perceived LT recipients’ cognition. We hypothesized that LT recipients with worse objective cognitive test scores or greater subjective cognitive decline reported by LT recipients or caregivers have worse symptom distress, poorer ability to perform household tasks, and decreased workplace productivity than those with better objective cognitive test scores or subjective cognitive status. This study further examined whether the above associations differ by post-transplant employment status because employment affects cognition (16).

## Patients and Methods

### Population

A convenience sampling was used in this cross-sectional single center study to recruit participants between December 2018 and September 2019. LT recipients were eligible to participate in this study if they were over 18 years old, had received a LT at least more than 3 months ago (to minimize the potential influences of early post-operative complications) but not more than 2 years post-transplant, had caregivers who could answer questions about the recipients’ cognition, and were able to speak and write English. LT recipients were excluded from this study if they had received any other organ transplant, such as kidney, had a history of a neurological disorder, such as Alzheimer’s disease or stroke, had a history of head injury, or were not able to provide informed consent. Caregivers identified by participating LT recipients were included in this study if they were over 18 years old and able to speak and write English. Those who were not able to provide informed consent were excluded from this study. Recruitment process of participants are described elsewhere (15), but in short, a total of 207 LT recipients were invited to participate in the study, and 60 provided written informed consent.

### Measures

LT recipients completed an online self-report survey assessing subjective cognition and patient-reported outcomes of physical and psychological symptom distress, ability to perform household tasks, and workplace productivity *via* Research Electronic Data Capture (REDCap) (17, 18). They then completed in-person, paper-and-pencil objective cognitive tests. Caregivers completed an online self-report survey assessing subjective cognition *via* REDCap.

#### Cognition

##### Subjective Cognition

The 39-item Modified Everyday Cognition (ECog) was used to assess LT recipient self-rated and caregiver-rated cognition in six domains: memory, language, visuospatial abilities, planning, organization, and divided attention (19, 20). LT recipients rated their current perceived difficulties in performing daily activities, and caregivers rated perceived difficulties that LT recipients currently have in performing daily activities from 1 (no difficulty) to 4 (severe difficulty/cannot do). “Don’t know” was available and scored as 0. Mean total scores that represent global subjective cognition and mean domain scores are available with higher scores indicating greater perceived difficulties in performing daily activities (19). The ECog was found to be valid in differentiating cognitive decline from normal cognition (19). Cronbach’s alpha coefficients in this study sample were good (0.89–0.98).

##### Objective Cognition

Global cognition was measured by the Repeatable Battery for the Assessment of Neuropsychological Status (RBANS) (21). The RBANS assesses five domains, attention, language, visuospatial/constructional abilities, and immediate and delayed memory, with 12 tests. Higher total scores indicate better global cognition (21). The RBANS has been shown to be effective in detecting mild cognitive impairment (22).

Cognition in three domains most commonly found to be impaired in the LT population (visuospatial abilities, executive function, and attention) (7, 23) were additionally assessed using reliable and valid cognitive tests. The Trail Making Test Parts A and B assess attention and executive function, respectively, and longer total time to complete the test indicates worse cognition (24). The Digit Span Backward test from the Neuropsychological Assessment Battery (NAB) assesses attention and executive function, and higher total scores are indicative of better cognition (25). The Rey-Osterrieth Complex Figure (Copy) assesses visuospatial constructional ability and executive function, and higher total scores are indicative of better cognition (26–28).

#### Patient-Reported Outcomes

##### Symptom Distress

The 28-item Rotterdam Symptom Checklist-Modified (RSCL-M) (29) asked LT recipients to rate the extent of physical symptom distress in the past week from 1 (Not at all) to 4 (Very much). Higher mean total scores indicate worse physical symptom distress (29). The validity of this questionnaire has been previously established (29). Cronbach’s alpha of this questionnaire was 0.89 in the current study. The 37-item Profile of Mood States-Short Form (POMS-SF) (30) asked LT recipients to rate their psychological distress in six subscales (depression, vigor, confusion, tension, anger, and fatigue) in the past 2 weeks from 0 (Not at all) to 4 (Extremely). Higher mean total scores indicate worse psychological distress (30). The validity of POMS-SF was established in various populations, including the kidney transplant population (30). Cronbach’s alpha of this questionnaire ranged from 0.82 to 0.95 in this study.

##### Ability to Perform Household Tasks

LT recipients completed the Creative Therapy Consultants (CTC) Homemaking Assessment, which was found to be reliable and valid to assess the performance of 29 household tasks in three categories: light (e.g., folding clothes), medium (e.g., washing dishes), and heavy (e.g., grocery shopping) tasks (31). Participants rated how much assistance they need to complete the tasks from 0 (Cannot complete without assistance) to 1 (Complete with no assistance). “Not applicable” was available if participants do not engage in certain tasks and scored as 0. Higher total weighted scores indicate higher household work productivity (32). Cronbach’s alpha for this questionnaire ranged between 0.84 and 0.92 in this study.

##### Workplace Productivity

LT recipients who were employed full-time or part-time at the time of survey administration completed the 25-item Work Limitations Questionnaire (WLQ) (33, 34). This questionnaire assesses the interference of health conditions in workplace productivity in four scales: Time, Physical, Mental-Interpersonal, and Output Demands. Participants rated difficulties at work in the past 2 weeks from 1 (None of the time) to 5 (All of the time) and rated their ability to work in the past 2 weeks from 1 (Able all of the time) to 5 (Able none of the time). “Does not apply to my job” was also available scoring as 0. Higher total scores indicate higher self-reported at-work productivity loss in the past 2 weeks (35). The WLQ has been validated with chronic disease populations (34). Cronbach’s alpha for this questionnaire ranged from 0.79 to 0.96 in this study.

#### Demographic and Clinical Characteristics

LT recipients and caregivers completed a self-report demographic questionnaire. Clinical characteristics of LT recipients, such as the date of transplant and Model for End Stage Liver Disease-Sodium (MELD) score (36), were extracted from medical records.

### Analysis

IBM SPSS Statistics version 26 (IBM, Armonk, NY, United States) and SAS Version 9.4 (SAS Institute, Cary, NC) were used to perform data analysis. Patient characteristics and scores for the study measures were described using means and standard deviations (SDs) for continuous measures and counts and proportions for categorical variables. Unadjusted and adjusted linear regression models were developed to examine the relationships of subjective and objective cognitive test scores with physical and psychological symptom distress and household tasks performance; adjusted models included LT recipients’ age, education, and months since LT. Only an unadjusted regression model was reported for workplace productivity due to the small number of subjects who were working either full- or part-time at the time of survey administration (*n* = 17). Among the four objective cognitive tests, only the RBANS that indicates global cognition was included in the models given the small sample size. For all models, the coefficient estimates were tabulated along with 95% confidence intervals (CI) and *p*-values. Correlations between subjective and objective cognitive domain scores and patient-reported outcomes were examined using Pearson’s correlation coefficient with 95% CIs to investigate which domains were specifically correlated with the outcomes. A two-tailed alpha of 0.05 was set as the level of statistical significance. Listwise deletion was used to deal with missing data.

This study performed additional analyses to examine differences in the findings by post-transplant employment status. Independent group t-tests, assuming unequal variance, were used to compare subjective and objective cognitive test scores and patient-reported outcomes of physical and psychological symptom distress and household tasks performance between employed and non-employed LT recipients. Unadjusted regression analyses and Pearson’s correlation coefficients, comparing the same variables as above but stratified by post-transplant employment status, were also performed.

## Results

### Participant Characteristics

Sixty pairs of LT recipients and their caregivers participated in this study. Table 1 summarizes the demographic of LT recipients. Most LT recipients were male (71.7%), white (93.3%), and married (71.7%). They had a mean age of 60.4 (SD = 6.9) and a mean of 14.1 (SD = 2.4) years of education, and mean time elapsed since LT was 12.9 months (SD = 7.0). The most common cause of liver failure was Non-alcoholic steatohepatitis (46.7%), and mean MELD score at LT was 21.7 (SD = 10.0; Table 1).

**TABLE 1:** Participant characteristics.

Characteristics	Frequency (%) or mean (SD)		
	All LT recipients (N = 60)	LT recipients Employed (N = 17)	LT Recipients Not Employed (N = 43)
Age (years)	60.4 (6.9)	57.9 (5.9)	61.5 (7.0)
Sex			
Male	43 (71.7%)	12 (70.6%)	31 (72.1%)
Female	17 (28.3%)	5 (29.4%)	12 (27.9%)
Race			
Black	2 (3.3%)	0 (0.0%)	2 (4.7%)
White	56 (93.3%)	16 (94.1%)	40 (93.0%)
Other (e.g., aboriginal)	2 (3.3%)	1 (5.9%)	1 (2.3%)
Marital Status			
Single	10 (16.7%)	2 (11.8%)	8 (18.6%)
Single, living with partner	3 (5.0%)	2 (11.8%)	1 (2.3%)
Married	43 (71.7%)	12 (70.6%)	31 (72.1%)
Widowed/separated	4 (6.7%)	1 (5.9%)	3 (7.0%)
Education	14.1 (2.4)	14.5 (3.0)	13.9 (2.1)
Household income			
$20,000 or less	10 (20.0%)	0 (0.0%)	10 (27.7%)
$20,001 to $40,000	9 (18.0%)	1 (7.1%)	8 (22.2%)
$40,001 to $60,000	10 (20.0%)	2 (14.2%)	8 (22.2%)
Over $60,000	21 (42.0%)	11 (78.6%)	10 (27.8%)
Insurance			
Government insurance	23 (38.3%)	2 (11.8%)	21 (48.8%)
Non-government insurance	32 (53.3%)	13 (76.5%)	19 (44.2%)
Multiple	4 (6.7%)	1 (5.9%)	3 (7.0%)
None	1 (1.7%)	1 (5.9%)	0 (0.0%)
Cause of liver disease			
Non-alcoholic steatohepatitis	28 (46.7%)	6 (35.3%)	22 (51.2%)
Alcohol	10 (16.7%)	2 (11.8%)	8 (18.6%)
Hepatitis C	11 (18.3%)	5 (29.4%)	6 (14.0%)
Autoimmune/cholestatic	7 (11.7%)	3 (17.6%)	4 (9.3%)
Others	4 (6.7%)	1 (5.9%)	3 (7.0%)
History of pre-transplant hepatic encephalopathy			
No	17 (28.3%)	9 (52.9%)	8 (18.6%)
Yes	43 (71.7%)	8 (47.1%)	35 (81.4%)
Time since transplant (months)	12.9 (7.0)	11.8 (6.8)	13.4 (7.1)
MELD score	21.7 (10.0)	13.6 (6.0)	24.9 (9.4)

Seventeen of 60 LT recipients were employed full time (*n* = 15, 25.0%) or part time (*n* = 2, 3.3%) after LT. While the characteristics of employed were generally comparable to non-employed LT recipients, employed recipients were relatively younger (mean age = 57.9, SD = 5.9 versus mean age = 61.5, SD = 7.0) and had a higher annual household income (annual household income over $60,000: 78.6% *versus* 27.8%). A smaller number of employed recipients had pre-transplant hepatic encephalopathy (47.1% *versus* 81.4%) and they had a lower MELD score at LT than the non-employed recipients (mean = 13.6, SD = 6.0 versus mean = 24.9, SD = 9.4; Table 1).

Most caregivers participated in this study were the spouse or significant other (73.3%) of LT recipients with a mean of 35 (SD = 12.6) years length of relationship. They tended to be female (80.0%), white (94.9%), and had a mean age of 57.1 (SD = 11.8) years and a mean of 13.9 (SD = 2.1) years of education. They reported that they spend a mean of 107 (SD = 56.8) hours per week with LT recipients.

### Subjective and Objective Cognition

The ECog scores of LT recipients are summarized in Table 2. Summaries of caregivers’ ECog scores were reported elsewhere (15) but briefly, scores of both LT recipients’ and caregivers’ ECog indicate mild perceived difficulties in performing daily activities (mean = 1.5, SD = 0.5; mean = 1.4, SD = 0.5).

**TABLE 2:** Summary of subjective and objective cognition, symptom distress, household tasks performance, and workplace productivity.

	All LT recipients (N = 60)	LT recipients employed (N = 17)	LT recipients not employed (N = 43)	*p*-value^a^
	Mean (SD)	Mean (SD)	Mean (SD)	
Ecog				
Global	1.5 (0.5)	1.2 (0.3)	1.6 (0.5)	**0.002**
Memory	1.7 (0.5)	1.4 (0.4)	1.8 (0.5)	**0.004**
Language	1.5 (0.6)	1.2 (0.3)	1.6 (0.6)	**0.004**
Visuospatial abilities	1.2 (0.4)	1.0 (0.1)	1.3 (0.5)	**0.000**
Planning	1.3 (0.5)	1.2 (0.3)	1.4 (0.5)	0.076
Organization	1.5 (0.7)	1.2 (0.3)	1.7 (0.7)	**0.001**
Divided attention	1.5 (0.7)	1.4 (0.6)	1.6 (0.7)	0.290
RBANS^b^				
Global	194.0 (25.5)^c^	210.9 (14.7)^d^	187.5 (25.9)^e^	**<0.0001**
Immediate memory index	40.1 (7.7)^c^	44.8 (5.0)^d^	39.3 (8.1)^e^	**0.003**
Visuospatial/constructional index	31.1 (4.5)^c^	32.3 (3.3)^d^	30.7 (4.8)^e^	0.154
Language index	28.6 (4.5)^c^	29.7 (4.6)^d^	28.1 (4.4)^e^	0.251
Attention index	48.9 (11.5)^c^	56.4 (6.8)^d^	46.0 (11.7)^e^	**0.000**
Delayed memory index	44.6 (5.7)^c^	47.6 (4.1)^d^	43.4 (5.9)^e^	**0.004**
Trail Making Test				
Part A	35.8 (17.1)^c^	29.1 (7.4)^d^	38.4 (19.1)^e^	**0.010**
Part B	90.0 (45.8)^c^	67.0 (16.5)^d^	98.9 (50.4)^e^	**0.001**
NAB Digit Span Backward	4.2 (2.0)^c^	4.9 (1.5)^d^	4.0 (2.0)^e^	0.071
Rey-Osterrieth Complex Figure (Copy)	27.6 (4.9)^c^	29.8 (4.3)^d^	26.7 (4.9)^e^	**0.025**
RSCL-M	1.6 (0.4)^c^	1.6 (0.5)^d^	1.6 (0.3)^e^	0.833
POMS-SF				
Total score	0.8 (0.5)	0.9 (0.5)	0.8 (0.5)	0.527
Depression	0.5 (0.7)	0.5 (0.7)	0.5 (0.7)	0.920
Vigor	1.6 (1.0)	1.9 (1.0)	1.6 (1.0)	0.289
Confusion	0.6 (0.6)	0.4 (0.6)	0.7 (0.6)	0.209
Tension	0.6 (0.7)	0.7 (0.7)	0.6 (0.7)	0.687
Anger	0.5 (0.6)	0.6 (0.6)	0.4 (0.6)	0.240
Fatigue	1.0 (1.0)	1.2 (1.1)	1.0 (0.9)	0.505
CTC Homemaking Assessment	90.5 (21.6)^f^	97.3 (7.9)	87.5 (24.9)^g^	**0.031**
WLQ				
Total Productivity Loss Score		3.4 (4.8)^d^		
Time Scale		17.0 (26.3)^d^		
Physical Scale		16.9 (24.1)^d^		
Mental-Interpersonal Scale		8.0 (14.2)^d^		
Output Scale		12.8 (21.5)^d^		

Note: Bold values denote statistical significance at the *p* < 0.05 level.

^a^Comparisons of cognition, symptom distress, household tasks performance, and workplace productivity between employed and non-employed LT recipients.

^b^Scores presented as raw scores.

^c^n = 57.

^d^ n = 16.

^e^ n = 41.

^f^ n = 56.

^g^ n = 39.

See Table 2 for the summary of objective cognitive performance.

#### Cognition and Post-Transplant Employment Status

Compared to LT recipients who did not return to work, employed LT recipients had lower global ECog scores (indicating less subjective concerns about cognition) and higher scores on global objective cognition and multiple domains (indicating higher levels of objective cognition; Table 2).

### Patient-Reported Outcomes

Summaries of patient-reported outcomes are described in Table 2. Scores of the RSCL-M and the POMS-SF indicated that LT recipients in this study have mild physical (mean = 1.6, SD = 0.4) and psychological symptom distress (mean = 0.8, SD = 0.5). Scores of the CTC Homemaking Assessment indicate that LT recipients could perform approximately 90% (SD = 21.6) of household tasks (Table 2).

#### Patient-Reported Outcomes and Post-Transplant Employment Status

LT recipients who returned to work after LT reported that they experience 3.4% (SD = 4.8) of productivity loss at work (Table 2). While the time scale score was the highest (mean = 17.0, SD = 26.3) indicating that recipients perceive the greatest difficulties handling time and scheduling demands at work, the mental-interpersonal scale score was the lowest (mean = 8.0, SD = 14.2) indicating that recipients perceive the lowest difficulties when performing tasks that require cognitive or social skills at work. Employed LT recipients had higher CTC Homemaking Assessment scores than non-employed recipients, while no significant differences were found in RCSL-M and POMS-SF scores (Table 2).

### Associations Between Subjective Cognition and Patient-Reported Outcomes

Higher total ECog scores of LT recipients were significantly associated with higher RSCL-M scores ( β = 0.30, *p* = 0.006; Table 3). Domain analysis revealed that ECog scores in all domains except for visuospatial abilities were fair to moderately associated with RSCL-M scores (*r* = 0.40–0.58; Table 4). Higher total ECog scores of caregivers were significantly associated with lower CTC Homemaking Assessment scores ( β = −18.55, *p* = 0.015; Table 3). Specifically, their ECog scores in memory and language were negatively correlated to CTC scores (*r* = −0.28 and −0.34, respectively; Table 4).

**TABLE 3:** Associations between subjective and objective cognition and symptom distress, household tasks performance, and workplace productivity.

	Unadjusted			Adjusted^a^		
	N	β (95% CI)	*p*-value	N	β (95% CI)	*p*-value
RSCL-M						
LT recipient ECog	60	0.39 (0.22, 0.57)	**<0.0001**	54	0.30 (0.09, 0.51)	**0.006**
Caregiver ECog	60	0.29 (0.10, 0.48)	**0.004**		0.14 (−0.08, 0.36)	0.213
LT recipient RBANS	57	−0.00 (−0.01, 0.00)	0.172		0.00 (−0.00, 0.01)	0.661
POMS-SF						
LT recipient ECog	60	0.27 (0.02, 0.52)	**0.035**	54	0.25 (−0.04, 0.55)	0.091
Caregiver ECog	60	0.27 (0.01, 0.52)	**0.040**		0.18 (−0.13, 0.49)	0.245
LT recipient RBANS	57	0.00 (−0.00, 0.01)	0.255		0.00 (−0.00, 0.01)	0.210
CTC Homemaking Assessment						
LT recipient ECog	56	−7.34 (−19.20, 4.51)	0.220	50	2.09 (−12.54, 16.71)	0.775
Caregiver ECog	56	−13.24 (−24.89, −1.60)	**0.027**		−18.55 (−33.38, −3.73)	**0.015**
LT recipient RBANS	53	0.01 (−0.24, 0.27)	0.916		−0.26 (−0.55, 0.04)	0.090
WLQ^b^						
LT recipient ECog	16	14.85 (10.40, 19.30)	**<0.0001**			
Caregiver ECog	16	4.92 (−3.09, 12.94)	0.209			
LT recipient RBANS	15	−0.13 (−0.31, 0.05)	0.129			

Note: Bold values denote statistical significance at the *p* < 0.05 level.

^a^ Adjusted for LT recipients’ age, education, and months since transplant.

^b^ Adjusted model for workplace productivity was not possible due to the small sample size (*n* = 16).

**TABLE 4:** Correlations between the domains of subjective cognition and symptom distress, household tasks performance, and workplace productivity.

		RSCL-M		POMS-SF		CTC homemaking assessment		WLQ^a^	
		r (95% CI)	*p*-value	r (95% CI)	*p*-value	r (95% CI)	*p*-value	r (95% CI)	*p*-value
LT recipient ECog (*n* = 60)	Memory	0.58 (0.38, 0.72)	**<0.0001**	0.31 (0.06, 0.53)	**0.014**	−0.28^b^ (−0.51, −0.02)	**0.032**	0.86 (0.63, 0.95)	**<0.0001**
	Language	0.40 (0.17, 0.60)	**0.001**	0.10 (−0.16, 0.34)	0.452	−0.21^b^ (−0.45, 0.06)	0.123	0.68 (0.28, 0.88)	**0.002**
	Visuospatial abilities	0.17 (−0.09, 0.40)	0.203	0.02 (−0.24, 0.27)	0.879	−0.00^b^ (−0.27, 0.26)	0.977	0.11 (−0.41, 0.57)	0.688
	Planning	0.48 (0.25, 0.65)	**<0.0001**	0.34 (0.09, 0.55)	**0.007**	−0.04^b^ (−0.30, 0.22)	0.742	0.83 (0.56, 0.94)	**<0.0001**
	Organization	0.45 (0.22, 0.63)	**0.0002**	0.31 (0.06, 0.52)	**0.015**	−0.05^b^ (−0.31, 0.22)	0.730	0.66 (0.24, 0.87)	**0.004**
	Divided attention	0.50 (0.28, 0.67)	**<0.0001**	0.39 (0.15, 0.59)	**0.002**	−0.19^b^ (−0.43, 0.08)	0.168	0.87 (0.66, 0.96)	**<0.0001**
Caregiver ECog (*n* = 60)	Memory	0.42 (0.18, 0.61)	**0.001**	0.25 (0.00, 0.48)	0.050	−0.28^b^ (−0.51, −0.02)	**0.033**	0.19 (−0.34, 0.63)	0.469
	Language	0.33 (0.09, 0.54)	**0.008**	0.15 (−0.11, 0.39)	0.240	−0.34^b^ (−0.55, −0.08)	**0.010**	0.31 (−0.22, 0.70)	0.228
	Visuospatial abilities	0.11^c^ (−0.16, 0.36)	0.435	0.10^c^ (−0.17, 0.35)	0.474	−0.17^d^ (−0.42, 0.11)	0.230	0.29 (−0.24, 0.69)	0.267
	Planning	0.28^e^ (0.02, 0.50)	**0.034**	0.30^e^ (0.04, 0.52)	**0.022**	−0.22^f^ (−0.46, 0.05)	0.103	0.26 (−0.27, 0.67)	0.312
	Organization	0.31^g^ (0.04, 0.54)	**0.023**	0.22^g^ (−0.06, 0.46)	0.115	−0.26^h^ (−0.50, 0.02)	0.064	0.33 (−0.24, 0.73)	0.233
	Divided attention	0.38^e^ (0.14, 0.58)	**0.003**	0.35^e^ (0.10, 0.55)	**0.007**	−0.26^f^ (−0.50, 0.01)	0.053	0.40 (−0.12, 0.75)	0.112

Note: Bold values denote statistical significance at the *p* < 0.05 level.

^a^n = 16.

b n = 56.

c n = 55.

d n = 53.

e n = 58.

f n = 54.

g n = 52.

h n = 50.

Higher LT recipients’ total ECog scores were significantly associated with higher WLQ scores ( β = 14.85, *p* < 0.0001; Table 3). Particularly, ECog scores in the domains of memory, language, planning, organization, and divided attention were correlated with higher scores on the WLQ (*r* = 0.66–0.87; Table 4). Caregiver ECog scores were not related to the WLQ, regardless of domain (Tables 3, 4). Note, these associations were unadjusted for LT recipients’ age, education, and months since transplant due to a small sample size.

#### Associations Between Subjective Cognition and Patient-Reported Outcomes by Post-Transplant Employment Status

This study further examined unadjusted associations between subjective cognition and patient-reported outcomes stratified by post-transplant employment status (Tables 5, 6). In the employed group, higher LT recipient total ECog scores were associated with higher RSCL-M ( β = 1.05, *p* = 0.005) and POMS-SF ( β = 1.46, *p* = 0.0003; Table 5). Domain analysis revealed that higher ECog scores of LT recipient in the domains of memory, planning, organization, and divided attention moderately to strongly correlated with higher RSCL-M (*r* = 0.47–0.69) and POMS-SF (*r* = 0.50–0.83; Table 6). Higher total ECog scores of both LT recipient and caregiver were related to lower CTC scores ( β = −14.96, *p* = 0.028; β = −14.97, *p* = 0.009, respectively; Table 5). While LT recipient ECog scores in memory and divided attention were related to lower CTC scores (*r* = −0.50 and −0.77, respectively), all caregiver ECog domain scores except for memory were moderately correlated with CTC Homemaking Assessment scores (*r* = −0.58–−0.66; Table 6). In the unemployed group, higher LT recipient and caregiver total ECog scores were associated with higher RSCL-M scores ( β = 0.36, *p* = 0.0002; β = 0.35, *p* = 0.0003, respectively). Nearly all of the recipient and caregiver ECog domain scores, except visuospatial abilities, were fair to moderately correlated with higher RSCL-M scores (*r* = 0.44–0.58 in LT recipients and *r* = 0.41–0.60 in caregivers; Table 6). No statistically significant associations were noted with POMS-SF or CTC scores (Table 5).

**TABLE 5:** Unadjusted associations between subjective and objective cognition and symptom distress, household tasks performance, and workplace productivity stratified by post-transplant employment status.

	LT recipients employed			LT recipients not employed		
	N	β (95% CI)	*p*-value	N	β (95% CI)	*p*-value
RSCL-M						
LT recipient ECog	17	1.05 (0.37, 1.73)	**0.005**	43	0.36 (0.18, 0.54)	**0.0002**
Caregiver ECog	17	−0.04 (−0.82, 0.74)	0.917	43	0.35 (0.17, 0.53)	**0.0003**
LT recipient RBANS	16	−0.02 (−0.03, −0.00)	**0.018**	41	−0.00 (−0.01, 0.00)	0.504
POMS-SF						
LT recipient ECog	17	1.46 (0.80, 2.11)	**0.000**	43	0.20 (−0.07, 0.48)	0.140
Caregiver ECog	17	0.55 (−0.29, 1.40)	0.184	43	0.27 (−0.00, 0.54)	0.052
LT recipient RBANS	16	−0.01 (−0.03, 0.01)	0.177	41	0.00 (−0.00, 0.01)	0.133
CTC Homemaking Assessment						
LT recipient ECog	17	−14.96 (−28.06, −1.86)	**0.028**	39	−3.31 (−19.26, 12.63)	0.676
Caregiver ECog	17	−14.97 (−25.64, −4.30)	**0.009**	39	−11.15 (−26.50, 7.20)	0.150
LT recipient RBANS	16	0.07 (−0.24, 0.38)	0.642	37	−0.09 (−0.44, 0.25)	0.588

Note: Bold values denote statistical significance at the *p* < 0.05 level.

**TABLE 6:** Correlations between the domains of subjective cognition and symptom distress, household tasks performance, and workplace productivity stratified by post-transplant employment status.

		RSCL-M		POMS-SF		CTC homemaking assessment	
		r (95% CI)	*p*-value	r (95% CI)	*p*-value	r (95% CI)	*p*-value
Employed (*n* = 17)							
LT recipient ECog	Memory	0.69 (0.32, 0.88)	**0.001**	0.74 (0.40, 0.90)	**0.0003**	−0.50 (−0.79, −0.02)	**0.035**
	Language	0.47 (−0.02, 0.77)	0.052	0.51 (0.04, 0.80)	**0.029**	−0.27 (−0.67, 0.24)	0.277
	Visuospatial abilities	0.04 (−0.45, 0.51)	0.876	0.16 (−0.35, 0.60)	0.528	−0.09 (−0.54, 0.41)	0.739
	Planning	0.63 (0.22, 0.85)	**0.004**	0.77 (0.46, 0.91)	**<0.0001**	−0.41 (−0.74, 0.09)	0.094
	Organization	0.54 (0.08, 0.81)	**0.021**	0.50 (0.03, 0.79)	**0.035**	−0.29 (−0.67, 0.23)	0.257
	Divided attention	0.47 (0.01, 0.78)	**0.048**	0.83 (0.58, 0.94)	**<0.0001**	−0.77 (−0.91, −0.47)	**<0.0001**
Caregiver ECog	Memory	−0.02 (−0.50, 0.46)	0.933	0.21 (−0.30, 0.63)	0.404	−0.37 (−0.72, 0.14)	0.139
	Language	−0.05 (−0.52, 0.44)	0.840	0.30 (−0.22, 0.68)	0.240	−0.60 (−0.84, −0.16)	**0.008**
	Visuospatial abilities	−0.10 (−0.56, 0.40)	0.693	0.23 (−0.28, 0.64)	0.372	−0.61 (−0.84, −0.18)	**0.006**
	Planning	−0.08 (−0.54, 0.41)	0.748	0.32 (−0.19, 0.70)	0.197	−0.58 (−0.83, −0.14)	**0.011**
	Organization	0.08^a^ (−0.45, 0.57)	0.771	0.33^a^ (−0.22, 0.72)	0.215	−0.58^a^ (−0.84, −0.10)	**0.018**
	Divided attention	−0.05 (−0.52, 0.44)	0.849	0.41 (−0.09, 0.74)	0.095	−0.66 (−0.87, −0.26)	**0.002**
Non-employed (*n* = 43)							
LT recipient ECog	Memory	0.58 (0.34, 0.75)	**<0.0001**	0.24 (−0.06, 0.51)	0.115	−0.21^b^ (−0.49, 0.12)	0.204
	Language	0.44 (0.16, 0.66)	**0.002**	0.06 (−0.25, 0.35)	0.711	−0.14^b^ (−0.44, 0.18)	0.378
	Visuospatial abilities	0.21 (−0.10, 0.48)	0.173	0.05 (−0.25, 0.35)	0.748	0.08^b^ (−0.24, 0.39)	0.611
	Planning	0.46 (0.19, 0.67)	**0.001**	0.27 (−0.03, 0.53)	0.075	0.04^b^ (−0.28, 0.35)	0.823
	Organization	0.50 (0.23, 0.70)	**0.0004**	0.36 (0.06, 0.59)	**0.018**	0.04^b^ (−0.28, 0.35)	0.787
	Divided attention	0.52 (0.25, 0.71)	**0.0003**	0.24 (−0.06, 0.51)	0.115	−0.09^b^ (−0.39, 0.23)	0.582
Caregiver ECog	Memory	0.60 (0.37, 0.76)	**<0.0001**	0.31 (0.01, 0.56)	**0.040**	−0.24^b^ (−0.52, 0.08)	0.135
	Language	0.48 (0.21, 0.68)	**0.001**	0.15 (−0.15, 0.43)	0.319	−0.28^b^ (−0.55, 0.03)	0.075
	Visuospatial abilities	0.16^c^ (−0.17, 0.46)	0.329	0.14^c^ (−0.19, 0.44)	0.413	−0.09^d^ (−0.41, 0.24)	0.595
	Planning	0.41^e^ (0.12, 0.64)	**0.007**	0.34^e^ (0.03, 0.58)	**0.030**	−0.16^f^ (−0.46, 0.17)	0.335
	Organization	0.41^f^ (0.10, 0.65)	**0.009**	0.22^f^ (−0.12, 0.50)	0.196	−0.20^g^ (−0.50, 0.14)	0.240
	Divided attention	0.58^e^ (0.34, 0.75)	**<0.0001**	0.37^e^ (0.07, 0.61)	**0.016**	−0.19^f^ (−0.49, 0.14)	0.252

Note: Bold values denote statistical significance at the *p* < 0.05 level.

^a^ n = 15.

^b^ n = 39.

^c^ n = 38.

^d^ n = 36.

^e^ n = 41.

^f^ n = 37.

^g^ n = 35.

### Associations Between Objective Cognition and Patient-Reported Outcomes

Objective test scores of LT recipients were not associated with any patient-reported outcomes within the entire sample (Table 3). In employed recipients, higher global cognition (RBANS total score) was related to lower RSCL-M scores (β = −0.02, *p* = 0.018; Table 5).

## Discussion

This cross-sectional study, to our knowledge, is the first to examine relationships between subjective and objective cognition and patient-reported outcomes of physical and psychological symptom distress, ability to perform household tasks, and workplace productivity among LT recipients. We found higher LT recipient self-rated cognitive decline was associated with higher physical symptom distress and workplace productivity loss. Higher caregiver-rated cognitive decline was associated with lower household tasks performance. However, these associations appear to be related to post-transplant employment status of LT recipients (employed vs. not employed). Cognitive decline measured by objective cognitive tests was not significantly associated with any of the patient-reported outcomes. These findings may suggest that LT recipients’ quality of life can be assessed with markers of subjective cognition, regardless of objective cognition.

Cognitive decline is one of the major health issues among LT recipients. Cognition, however, is not regularly assessed in transplant practice due to multiple reasons, including lengthy cognitive tests. Subjective cognition that is easily assessed by a valid and reliable self-report survey has potential to be used as a proxy for objective cognition at a busy transplant clinic. Most participants completed the ECog within 2 min (15). While we have shown that there are fair to moderate correlations between objective and subjective cognition among LT recipients (15), the present study highlights that these different cognitive measures may provide complementary information, particularly regarding patient-reported outcomes that require cognitive skills, such as workplace productivity.

Subjective cognition, not objective cognition, was associated with LT recipients’ perceived health-related quality of life and independence. While objective cognitive decline may contribute to develop worse clinical outcomes, such as graft failure and mortality (37), subjective cognitive decline may affect LT recipients’ overall quality of life (38). Recipient self-rated cognition, however, was not associated with psychological symptom distress. This finding was inconsistent with the literature in general (39) and chronic illness populations (40) that documented strong relationships between subjective cognitive decline and psychological symptoms. This unexpected finding may be related to the types of psychological symptoms assessed in this study. This study assessed overall mood state by measuring six different dimensions of mood (depression, vigor, confusion, tension, anger, and fatigue), while many previous studies were limited to the symptoms of depression and/or anxiety (39, 40). Additionally, this study recruited LT recipients regardless of their subjective cognitive status as opposed to intentionally recruiting individuals with cognitive complaints (39). Replication with a larger sample size that intentionally recruit LT recipients with cognitive complaints may advance the understanding of the associations between subjective cognition and psychological symptom distress in different dimensions of mood.

LT recipient self-rated cognition, not caregiver-rated cognition, was significantly correlated with physical symptom distress, indicating that LT recipients who experience subjective cognitive decline may also be experiencing higher levels of physical symptom distress. While the relationship between subjective cognition and physical symptoms, such as fatigue, has been documented in the chronic illness literature (41), the mechanism underlying this relationship has not been thoroughly examined. One possibility is that LT recipients who have subjective cognitive decline in memory and executive function, essential cognitive domains to self-manage, may believe that they are not capable to properly manage their physical symptoms. Their belief may reduce engagement in symptom monitoring and management, resulting in increased symptom distress.

Findings from this study may contribute to understanding the low employment rates among LT recipients. Consistent to previous studies in chronic disease populations (42), this study found a strong relationship between LT recipients’ subjective cognitive decline and self-reported workplace productivity loss. Particularly, LT recipients’ subjective ratings on memory and executive function, the core cognitive skills that are essential to perform job tasks, were correlated with the workplace productivity loss. Although these findings should be understood with caution due to a small sample size of employed LT recipients, they may suggest that subjective cognitive decline could be one of the barriers that prevent LT recipients from returning to work. Participating in work after LT is crucial to maintain independent and productive lives and reduce burden on recipients’ families and communities. Nevertheless, fewer than 25% of LT recipients are employed in paid work within 2 years post-transplant (43, 44). Findings of this study suggest an in-depth examination of the impacts of subjective and objective cognition on employment rates and workplace productivity to tackle unemployment among LT recipients.

Finally, this study provides insight into to the planning of individualized treatment to advance patient’s quality of life. Caregivers seem to be more accurate than patients themselves in estimating cognitive changes (15, 45). Their subjective ratings of LT recipients’ cognition may provide essential information regarding functional independence of LT recipients that can be used in the development of an individualized rehabilitation therapy. Further, employment status seems alter the associations between subjective cognition and patient-reported outcomes. While employed LT recipients have better subjective and objective cognition than non-employed recipients, their subjective cognition is more broadly associated with patient-reported outcomes than non-employed recipients’ subjective cognition. Such findings may imply that psychosocial circumstances of recipients, such as employment status, should be considered when planning a treatment for LT recipients.

A novel finding of this study is that subjective cognitive decline, not objective cognitive decline, is associated with LT recipients’ overall wellbeing. This study also suggests subjective cognition could be associated with low employment rate in this population. However, a few limitations of this study should be noted. Because of the cross-sectional nature of this study, the observed associations do not indicate cause and effect. The small sample recruited at a single center limits the generalizability of the study findings. Particularly, findings related to post-transplant employment status should be understood with caution due to the small sample size of employed LT recipients. Potential confounders that may affect the patient-reported outcomes, such as comorbidities and employment status before LT, were not included in the analysis.

In conclusion, LT recipients with subjective cognitive decline may benefit from extra support on improving their quality of life. In practice, clinicians may consider paying attention to LT recipients’ complaints about their cognition as they may reflect LT recipients’ poor quality of life. Clinicians may also consider including caregivers when developing an individualized post-LT care plan as these caregivers may provide supplemental information regarding LT recipients’ cognition and functional independence. Future longitudinal studies with a larger diverse sample are suggested to investigate the underlying mechanism of the relationships between subjective cognition and patient-reported outcomes. Such findings may contribute to identify strategies to support recipients with subjective cognitive decline to optimize their health and retain independence after LT.

## Data Availability

The raw data supporting the conclusion of this article will be made available by the authors, without undue reservation.

## Abbreviations

CI, confidence intervals; CTC, creative therapy consultants; ECog, everyday cognition; LT, liver transplant; MELD-Na, model for end-stage liver disease with sodium; NAB, neuropsychological assessment battery; POMS-SF, profile of mood states-short form; RBANS, repeatable battery for the assessment of neuropsychological status; REDCap, research electronic data capture; RSCL-M, rotterdam symptom checklist-modified; SD, standard deviation; WLQ, work limitations questionnaire.
